# Functional Identification of MhPYL4 Involved in Iron-Deficiency Stress in Malus Halliana Koehne

**DOI:** 10.3390/plants13162317

**Published:** 2024-08-20

**Authors:** Xiaoya Wang, Zhongxing Zhang, Yongjuan Dong, Yanxiu Wang

**Affiliations:** College of Horticulture, Gansu Agricultural University, Lanzhou 730070, China; 18095526010@163.com (X.W.); 18394133890@163.com (Z.Z.); dongyj20231202@163.com (Y.D.)

**Keywords:** *MhPYL4* gene, molecular cloning, expression analysis, Fe-deficiency stress

## Abstract

The PYL protein family are crucial sensors of the core signals of abscisic acid (ABA) and significantly influence the plant’s response to ABA-mediated abiotic stresses as well as its growth and development. However, research on the role of the *MhPYL4* gene in iron (Fe) deficiency in apple trees is limited. Studies have shown that the *MhPYL4* gene, when exposed to Fe-deficiency stress, exhibits more rapid transcriptional upregulation than other genes’ quickly elevated transcription. However, the precise mechanism by which it alleviates this stress remains unclear. The *MhPYL4* gene (ID:103432868), isolated from *Malus halliana*, was analyzed to elucidate its function. *Arabidopsis* plants engineered to overexpress the *MhPYL4* gene exhibited increased leaf chlorosis and slower growth in response to Fe stress compared to the unmodified controls. The transgenic plants also exhibited elevated levels of superoxide dismutase (SOD), peroxidase (POD), and catalase (CAT) activities, as well as ferric chelate reductase (FCR) activities. Levels of malondialdehyde (MDA), hydrogen peroxide (H_2_O_2_), and superoxide anion (O_2_^−^) were increased. In addition, these transgenic plants had lower concentrations of proline (Pro) and Fe^2+^, which indicated that their stress tolerance was reduced. Similarly, the overexpression of *MhPYL4* in apple calli resulted in inhibited growth and increased susceptibility under Fe stress conditions. Physiological evaluations indicated that the overexpression of *MhPYL4* in *Arabidopsis* reduced its Fe stress tolerance by inhibiting chlorophyll synthesis. In apple calli, it altered pH levels, antioxidant enzyme activity, and Fe-reducing capabilities under the same stress conditions. In summary, the elevated expression of the *MhPYL4* gene reduced the tolerance of both *Arabidopsis* and apple calli to Fe stress, suggesting that *MhPYL4* acts as a negative regulator in response to Fe deficiency.

## 1. Introduction

Iron (Fe) is an essential element in coenzymes involved in electron transfer reactions and is essential for various physiological processes in plants, such as energy production, photosynthesis, and hormone biosynthesis [1]. Due to its low solubility in soil and susceptibility to redox reactions, the transportation of Fe within plants requires binding to a suitable chelator [2]. Plants uptake Fe^2+^ through their roots and transport it to different tissues and organs to support growth and development [3]. However, despite the abundance of Fe in soils, plants often struggle to effectively utilize it, especially in alkaline soils and oxygen-deprived conditions, where insoluble complexes impede root absorption [4]. Symptoms of Fe deficiency in plants manifest as leaf chlorosis and the loss of green pigmentation between veins, potentially leading to plant death in severe cases [5]. In the natural environment, a combination of factors prompts an integrated plant response. Coordination is achieved by key protein–regulator complexes that interconnect various cellular signaling pathways. Imbalances in iron levels can impair plant growth and their capacity to handle abiotic stresses [6].

The ABA signaling pathway plays a critical role in enhancing plant resistance to osmotic stresses, such as drought and salinity [7]. The PYR/PYL/PCAR gene family encodes ABA receptors, thus acting as a crucial mediator of abscisic acid core signals in response to abiotic stress, particularly drought. Overexpression of PYL/PYR proteins has been demonstrated to enhance drought tolerance in crops by increasing ABA sensitivity [8]. Furthermore, upregulation of specific genes within this protein family, such as *AtPYL5* and *TaPYR1*, can boost drought resistance by suppressing ABA signaling activation and elevating osmotic substance content and enzyme activities [9]. Research indicates a significant increase in the transcript abundance of PYR family in response to drought and salt stress, aiding in plant stress regulation by strengthening the ABA binding ability [10]. Most studies on PYL have focused on *Arabidopsis* thaliana and rice, which are model plants and major crops, respectively [11]. However, there is limited information on the involvement of PYL in signal transduction for apple resistance to abiotic stresses, including Fe deficiency and salinity. Therefore, investigating the role of PYL in apple could lay the foundation for molecular engineering to improve iron deficiency resistance in this crop.

*Malus halliana*, an apple rootstock indigenous to Gansu’s arid and saline lands, has been shown to possess excellent stress tolerance in various studies [12]. Despite limited research on this species in China, its potential for addressing challenges such as chlorosis disease due to Fe deficiency in apple trees is significant. In regions with high-pH calcareous soils, like China, traditional methods of increasing soil Fe content to alleviate deficiency may not be sustainable over the long term [13]. Therefore, the screening for genes, such as *MhPYL4*, involved in the response to Fe-deficiency stress presents a more efficient and effective solution for developing Fe-deficiency-resistant plants. Through gene cloning and functional characterization in both *Arabidopsis* and apple calli, the *MhPYL4* gene has shown promising results regarding its potential role in regulating responses to Fe-deficiency stress. While the exact mechanism of action of *MhPYL4* on Fe-deficiency stress is still unknown, its significant expression under such conditions suggests an importance in plant responses to nutrient deficiencies. By further exploring the functions of this gene and its potential applications in improving plant resilience to Fe deficiency, there is a potential for developing strategies that enhance the productivity and sustainability of fruit industries, particularly in regions facing similar soil nutrient challenges.

## 2. Materials and Methods

### 2.1. Plant Materials and Treatments

Wild-type *Arabidopsis* thaliana plants were grown in an MS medium (MS + 30 g/L sucrose + 8 g/L agar, pH = 5.8–6.0). They were vernalized at 4 °C for 3 days and then transferred to a 26 °C environment. Once green seedlings emerged, they were transplanted to a growth substrate consisting of peat, perlite, and vermiculite in a 3:1:1 ratio. They were used for *Arabidopsis thaliana* infestation during the blooming phase, with sessions held weekly for a total of 3–4 times. Transgenic *Arabidopsis* seeds were selected on a kanamycin-containing medium to ensure the homozygosity of the transgenic plants. The experiment involving Fe-deficiency-induced stress treatment was conducted in triplicate.

Wanglin apple calli were grown on an MS medium with 1.5 mg/L of 2,4-D and 0.4 mg/L of 6-BA every 20 days. After creating transgenic apple calli using the method established by Gao [14], transgenic and wild-type control apple calli were placed in an Fe-free MS medium (MS + 1.0 mg·L^−1^ 6-BA + 1.0 mg·L^−1^ 2,4-D + 30 g·L^−1^ sucrose + 7.0 g·L^−1^ Agar, pH 5.8–6.2) for a period of 20 days. Observations were then made, phenotypic changes were recorded, and data relating to the corpus callosum were measured.

A uniform 8-leaf *Malus halliana* seedling with no infection was selected and pre-cultured for 7 days on an Fe-sufficient (+Fe) MS medium for experiment. The Fe concentration was set to 0 μM Fe (−Fe) according to Han et al. [15]. Each treatment was repeated three times with five plants each.

### 2.2. Real-Time PCR

The total RNA was extracted from the samples using an RNA extraction kit from BioTeke Corporation in Beijing, China. Reverse transcription was carried out using TaKaRa’s PrimeScriptTM RT reagent kit with a gDNA Eraser (Perfect Real Time). Primers were designed with the assistance of Shanghai Sangon Biological Engineering Co., Ltd., and the sequences were obtained from the NCBI database (Supplementary Table S1). Real-time PCR primer pairs were selected based on the design and are listed in Table S1. In addition, the cDNA of *M. halliana* plantlets was regarded as the template and GAPDH as a reference for quantitative real-time PCR. Quantitative data analysis was conducted using the 2-Ct method. The reaction system consisted of TB GreenTM Premix Ex Taq II, 10 μmol of upstream and downstream primers each, the cDNA template, and dd H_2_O in dosages of 10, 1, 1, 2, and 6 μL, respectively. The reaction conditions involved an initial pre-denaturation at 95 °C for 3 min, followed by denaturation at 95 °C for 5 s, annealing at 56 °C for 30 s, and extension at 72 °C for 30 s, which was repeated for 40 cycles. Each sample was run in triplicate to ensure the reliability of the results.

### 2.3. Bioinformatics Analysis and Subcellular Localization of MhPYL4

The physical and chemical characteristics of the proteins were determined using the Protparam website (http://web.expasy.org/protparam/, accessed on 10 June 2024). Protein amino acid sequence alignments were conducted with DNAMANexe software version 10. Phylogenetic trees were generated using MEGAX, utilizing the neighbor-joining (NJ) method. The cis-acting components on the *MhPYL4* promoter were identified using PlantCARE. The subcellular localization of tobacco was carried out by the method of Sun et al. [16] (Supplementary Table S2).

### 2.4. MhPYL4 and MhPYL4-A Gene Cloning and Vector Construction

Leaf RNA was isolated using the Trizol method to determine reverse transcription and concentration. The coding sequence (CDS) of the *MhPYL4* gene and the antisense sequence (*MhPYL4*-A) were obtained from the NCBI database. Specific primers were designed with DNAMAN software. The gel-extracted product of the target gene was linked with pMD19-T and then introduced into *E. coli* for sequencing. The purified plasmid was combined with PRI-GFP through homologous recombination. Positive colonies were identified and sequenced, plasmids were extracted, and *Agrobacterium* tumefaciens LB4404 was transformed using the freeze–thaw method for subsequent gene transformation.

### 2.5. Agrobacterium-Mediated Transformation of Arabidopsis thaliana

Employing the genetic transformation technique as reported by Hu et al. [17], we acquired transgenic *Arabidopsis* seeds harboring the *MhPYL4* gene. The seeds were first treated with 75% ethanol for 5 min, followed by a 10 min treatment with 26% sodium hypochlorite. Afterwards, they were rinsed thoroughly 3 to 5 times with deionized water to remove residual chemicals. The seeds were then screened for resistance on a Murashige and Skoog (MS) medium containing 30 mg·L^−1^ kanamycin. The resistant plants were further grown on the MS medium with an increased concentration of 250 mg·L^−1^ kanamycin. This process was continued until T3 generation transgenic homozygous seeds were successfully obtained.

### 2.6. Agrobacterium-Mediated Transformation of Apple Calli

The suspension-cultured calli, which were in the exponential growth phase for about 2 weeks, were subcultured with *Agrobacterium*, and the bacterial solution was removed after 30 min, following the method of Li et al. [18]. The calli were transferred to an antibiotic-free solid medium for dark culture for 2 days and then washed with sterile water 3 to 5 times for 3 min each. Subsequently, the calli were transferred to a screen medium containing 250 mg·L^−1^ of cephalosporin and 30 mg·L^−1^ of kanamycin. Resistant calli were grown for 30 to 60 days. DNA extraction and real-time quantitative PCR were performed.

### 2.7. Treatment with Fe Deficiency in Transgenic Arabidopsis, and Apple Calli

First, the seeds of the wild type and of the homozygous transgenic *A. thaliana* of the T3 generation were disinfected and placed on an MS solid medium, vernalized at 4 °C for 3 days, and cultivated in a light incubator for 10 days. Subsequently, phenotypes were observed and physiological indices were determined by cultivating on an MS solid medium that was Fe-sufficient (+Fe:40 uM Fe) and Fe-deficient (−Fe:0 uM Fe) for 20 days in dark conditions at 25 °C.

Finally, overexpressed apple calli and wild-type calli were subcultured on an MS solid medium for 15 days, and then on an Fe-sufficient (+Fe) and Fe-deficient (−Fe) solid medium for 20 days. Phenotypes were observed and related indicators were measured. Fe was supplied in the form of Fe-EDTA.

### 2.8. Physiological Index Detection Measurement

*Arabidopsis thaliana* leaves were cultivated under iron-sufficient and iron-deficient conditions for a period of 20 days, where photosynthetic pigments were extracted by 80% acetone. Subsequently, the absorbance of the obtained extracts was determined by spectrophotometry at different wavelengths, including 470, 663, and 646 nm. The contents of photosynthetic pigments, including chlorophyll (Chla), chlorophyll (Chlb), chlorophyll a+b (Chla+b), and carotenoids (Car), were calculated as described by Silas et al. [19], and the Chla+b content was the sum of the Chla and Chlb contents. In addition, the activities of superoxide dismutase (SOD), peroxidase (POD), and catalase (CAT) were determined by spectrophotometry using a kit from Su Zhou Keming Biological (China). Finally, ferric reductase activity (FCR) activity was determined according to Schikora and Schmidt [20], with some modifications. The analysis of acidification was carried out according to the method of Zhao [21]. Additionally, endogenous hormones, including growth hormone (IAA) and abscisic acid (ABA), were determined following Sharma et al. [22].

### 2.9. Statistical Analysis

Experimental effects were tested by analysis of variance and comparisons of means were conducted with the Duncan’s test (*p* < 0.05). SPSS version 22.0 (IBM, Armonk, NY, USA) was used for statistical analyses, and Origin 8.0 software (Origin2018, Hampton, MA, USA) was used to process figures.

## 3. Results

### 3.1. Expression Patterns of Several PYL Genes in Response to Fe-Deficiency Stress

**Figure 1:**
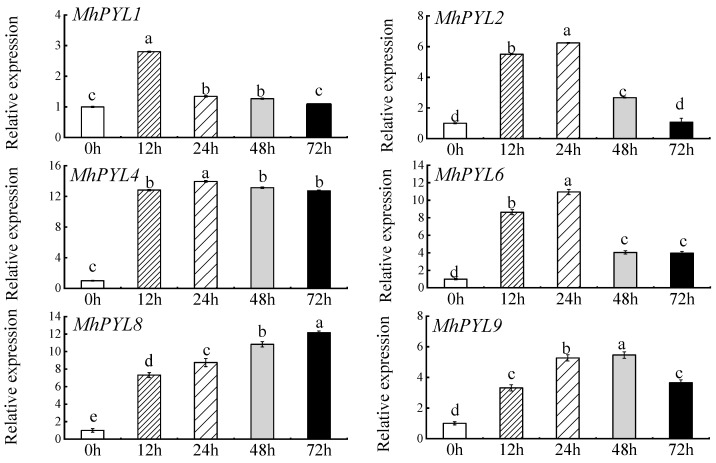
Expression levels of several *PYL* family genes on Fe-deficient medium at 0, 12, 24, 48, and 72 h in *Malus halliana*. Different letters above the bars indicate significant differences (*p* < 0.05) as assessed by one-way ANOVA and the LSD test (*p* < 0.05).

Utilizing the *Malus halliana* transcriptome database, six Fe-deficiency functional genes (PYL1, PYL2, PYL4, PYL6, PYL8, and PYL9) were chosen for qRT-PCR analysis. Figure 1 illustrates a significant increase in the expression of PYL4 at 12 h, maintaining stability until 72 h with a 13.12-fold increase compared to 0 h.

### 3.2. Analysis of the MhPYL4 Gene

**Figure 2:**
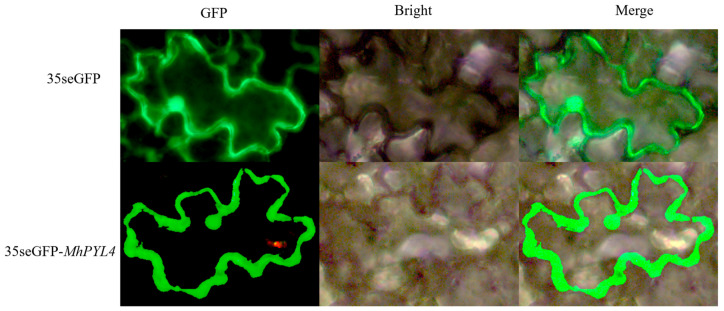
*MhPYL4* expression in tobacco cell localization analysis.

Using *Malus halliana* seedling cDNA as a template, a 624 bp *MhPYL4* band (Supplementary Figure S1A) and a 180 bp *MhPYL4-A* band (Supplementary Figure S1C) were obtained. Sequence analysis showed that these were *MhPYL4* (Supplementary Figure S1B) and *MhPYL4-A* (Supplementary Figure S1D), and both were successfully ligated into the pRI101 expression vector. Transgenic *MhPYL4* was used to obtain transgenic material, DNA extraction and validation of *Arabidopsis* (Supplementary Figure S1E), and apple calli (Supplementary Figure S1F). Transgenic *MhPYL4-A* was used to obtain apple calli transgenic material (Supplementary Figure S1G). As shown in Supplementary Table S3, the physical and chemical properties of the *MhPYL4* gene in *Malus halliana* showed that 207 amino acids were encoded, with the molecular weight of 22.74 kD. The lipid coefficient was 80.34. Furthermore, the positive and negative charges were 17 and 20, respectively, and the isoelectric point PI was 6.44, which is alkaline. The average hydrophilicity was 0.293, which is a hydrophilic protein. The instability coefficient was 47.65, indicating that the protein is unstable. Sequence alignment was performed to compare the amino acid sequences encoded by *MhPYL4* with those of other species, which showed high similarity at the C-terminal and some differences at the N-terminal (Supplementary Figure S1). In addition, the sequences of the *MhPYL4* protein and *MhPYL4* protein from other species were selected, and the phylogenetic tree was constructed by neighbor joining (NJ) using MEGA-X software v11.0.13 (Supplementary Figure S2). The result showed that *MhPYL4* is closely related to *Helianthus annuus* (XP_02202514.1). Finally, analysis of cis-acting elements on the *MhPYL4* promoter (Supplementary Figure S3) showed that the *MhPYL4* promoter sequence contains a variety of stress-related cis-regulatory elements, for example, the ABRE associated with ABA, the CGTCA motif associated with MeJA, the G-box associated with light, and the TCA element associated with salicylic acid. These results suggest that *MhPYL4* is regulated by abscisic acid, salicylic acid, light, and other environmental signals and is involved in a number of biological processes. To investigate the expression site of *MhPYL4*, we generated the subcellular localization vector pART-CAM-EGFP through homologous recombination. Upon injection into tobacco (Figure 2), our observations revealed that the green fluorescent fusion protein predominantly localized to the nucleus, with a minor presence in the cytoplasm.

### 3.3. Confirmation of Transgenic Arabidopsis thaliana and Overexpressed Apple Calli

**Figure 3:**
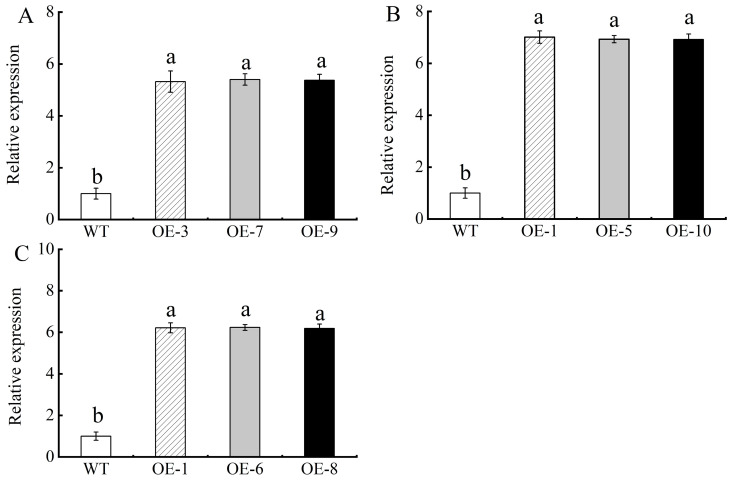
*MhPYL4* and *MhPYL4-A* transgene overexpression level. (**A**) *MhPYL4 Arabidopsis thaliana*. (**B**) *MhPYL4* overexpressed apple calli. (**C**) *MhPYL4-A* overexpressed apple calli. Note: error bars denote the SD of three replicates. Different letters above the bars indicated significant differences (*p* < 0.05) as assessed by one-way ANOVA and the least significant difference (LSD) test. The same below.

Using qRT-PCR, we measured the levels of gene *MhPYL4* in transgenic *Arabidopsis* and apple calli overexpressing *MhPYL4* and *MhPYL4*-A. Compared to control plants, we observed a higher expression of *MhPYL4* in transgenic *Arabidopsis* (Figure 3A) and apple calli overexpressing *MhPYL4* (Figure 3B), as well as in apple calli overexpressing *MhPYL4*-A (Figure 3C). These results suggest that overexpression of *MhPYL4* occurs in both *Arabidopsis* and apple calli. Additionally, *MhPYL4-*A exhibited increased expression in the overexpressed apple callii.

### 3.4. The PYL4-Overexpressing Arabidopsis Seedlings Display Enhanced Sensitivity to Fe Deficiency

**Figure 4:**
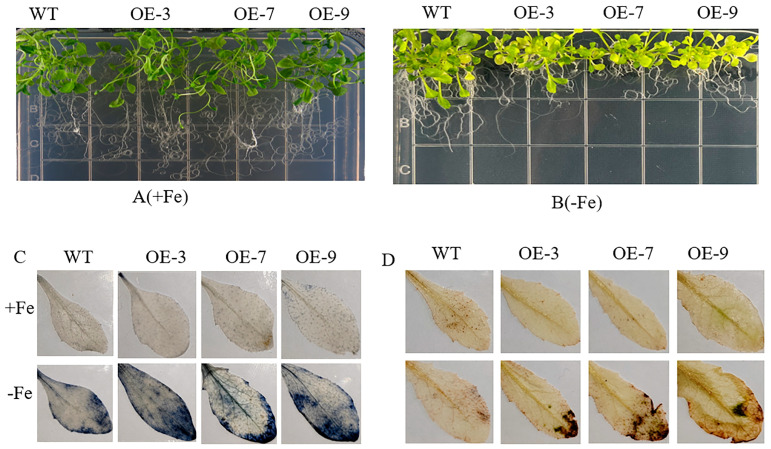
The phenotype, NBT and DAB staining of *MhPYL4-*OE and wild type. (**A**) The phenotypes of +Fe. (**B**) The phenotypes of −Fe (**C**) NBT staining. (**D**) DAB staining.

The results presented in Figure 4 demonstrate that both transgenic *Arabidopsis* and wild-type (WT) control *Arabidopsis* exhibited normal growth with green leaves when exposed to Fe-supplemented environments. In contrast, the transgenic lines showed more severe leaf chlorosis and reduced growth under Fe-deficiency stress compared to WT *Arabidopsis*. Additionally, the levels of chlorophyll a, chlorophyll b, chlorophyll a+b, and carotenoids in the transgenic lines (OE-3, OE-7, and OE-9) were not significantly different from those in the wild-type control under Fe-abundant conditions. However, a notable decrease in the content of photosynthetic pigments was observed in transgenic *Arabidopsis thaliana* under Fe-deficiency conditions (Supplementary Figure S5).

The O_2_^−^ and H_2_O_2_ content in the leaves of transgenic and WT *Arabidopsis* plants were analyzed using staining techniques with NBT and DAB. Ectopic expression of *MhPYL4* in *Arabidopsis* resulted in higher O_2_^−^ and H_2_O_2_ levels in the leaves under Fe-deficient conditions compared to the WT control. This indicates that transgenic plants facilitated ROS accumulation in the leaves, leading to increased cellular damage. Consequently, it was determined that overexpression of *MhPYL4* compromised *Arabidopsis* resistance to Fe-deficiency stress. These results suggest that the ectopic expression of *MhPYL4* significantly influences the plant’s response to Fe-deficiency stress. By elevating O_2_^−^ and H_2_O_2_ levels in the leaves, transgenic plants worsen cellular damage, diminishing their ability to withstand Fe-deficiency stress. Overall, these findings underscore the importance of maintaining appropriate ROS levels in plants to enhance their resilience to environmental stresses such as Fe deficiency.

**Figure 5:**
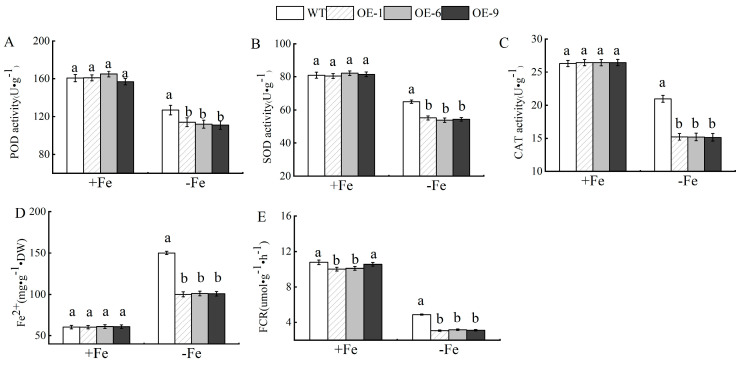
Overexpression of *MhPYL4 Arabidopsis* thaliana for resistance to Fe-deficiency stress. (**A**) POD activity. (**B**) SOD activity. (**C**) CAT activity. (**D**) Fe^2+^ content. (**E**) FCR activity. Different letters above the bars indicate significant differences (*p* < 0.05) as assessed by one-way ANOVA and the LSD test (*p* < 0.05).

In addition, the enzyme activities of SOD, POD, CAT, FCR, and in transgenic *Arabidopsis* plants were different from those of the wild type under Fe-deficiency stress (Figure 5), and their enzyme activities were significantly lower than those of the wild type.

### 3.5. Resistance of Transgenic MhPYL4 Apple Calli to Fe-Deficiency Stress

**Figure 6:**
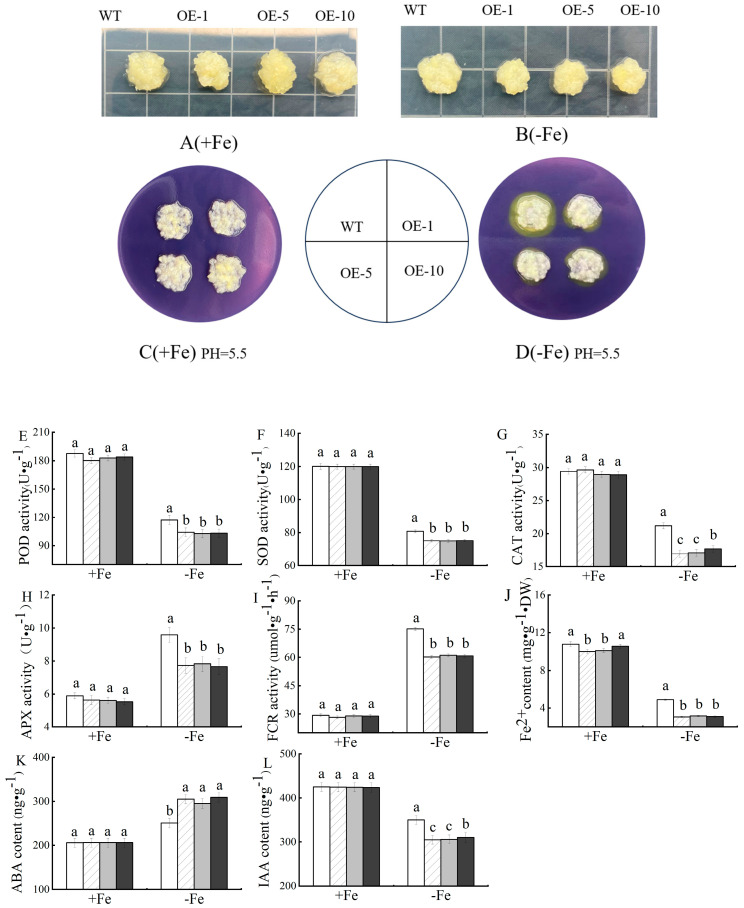
Phenotypes of *MhPYL4* transgenic and wild-type (WT) control apple calli grown on Fe-sufficient or Fe-deficient media for 20 days. (**A**) Fe-sufficient. (**B**) Fe-deficient. A yellow color around apple calli indicates acidification. (**C**) Fe-sufficient. (**D**) Fe-deficient. (**E**) Peroxidase (POD) activity. (**F**) Superoxide dismutase (SOD) activity. (**G**) Catalase (CAT) activity. (**H**) Ascorbate peroxidase (APX) activity. (**I**) Ferric reductase activity (FCR) activity. (**J**) Fe^2+^ content. (**K**) ABA content. (**L**) IAA content. Different letters above the bars indicate significant differences (*p* < 0.05) as assessed by one-way ANOVA and the LSD test (*p* < 0.05).

Figure 6 shows that, under Fe-sufficient conditions, the growth of overexpressed and wild-type (WT) apple calli was not significantly different. However, a noticeable distinction was observed under Fe-deficient conditions, with transgenic apple calli exhibiting slower growth and a smaller size compared to WT apple calli. Furthermore, the overexpressed lines OE-1, OE-5, and OE-10 displayed decreased enzyme activities, particularly in peroxidase (POD), superoxide dismutase (SOD), catalase (CAT), and ascorbate peroxidase (APX), as shown in Figure 6. Furthermore, measurements of ferric chelate reductase (FCR) activity and the content of divalent Fe^2+^ indicated lower values in apple calli overexpressing *MhPYL4* than in the wild-type (WT) calli after exposure to Fe-deficiency stress. These results collectively indicate that overexpressing the *MhPYL4* gene increases the susceptibility of apple plants to Fe-deficiency stress.

After exposure to Fe-deficiency stress, analysis of the endogenous plant hormones ABA and IAA (as shown in Figure 6) showed minimal changes in the levels of IAA and ABA in both transgenic and wild-type calli under normal growth conditions. However, when exposed to Fe-deficiency stress, transgenic calli had a lower IAA content compared to the WT, while the ABA content was higher in the transgenic apple calli than in the WT. This suggests that the introduction of *MhPYL4* may enhance apple Fe-deficiency tolerance by either inhibiting IAA-related synthase activity or upregulating the expression of ABA-related synthase genes. Furthermore, staining with bromocresol violet, which appeared as yellow, indicated that *MhPYL4* transgenic calli released fewer H^+^ into the medium under Fe-deficiency conditions when compared to the WT control (Figure 6).

### 3.6. Resistance of Transgenic MhPYL4-A Apple Calli to Fe-Deficiency Stress

**Figure 7:**
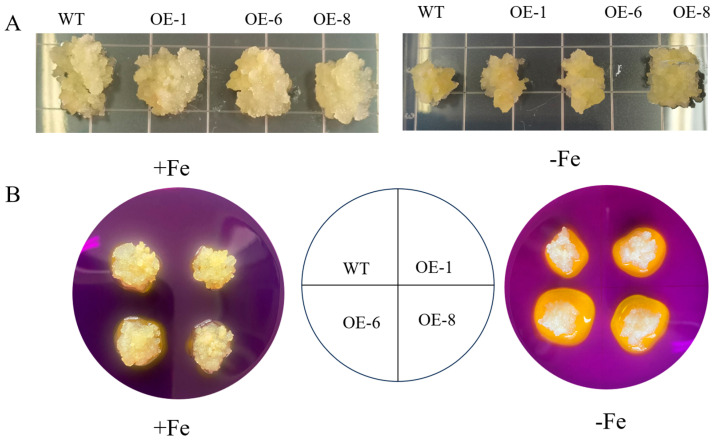
The phenotypes of apple calli overexpressing *MhPYL4*-A and the wild-type (WT) control, cultivated on Fe-sufficient or Fe-deficient media for 15 days, were evaluated through acidification analysis using a medium containing the pH indicator dye bromocresol violet. (**A**) *MhPYL4*-A lines. (**B**) *MhPYL4*-A apple calli lines.

According to the findings presented in Figure 7, there was no significant discrepancy in growth status between apple calli that overexpressed specific genes and regular apple calli when subjected to Fe-rich environments. Nevertheless, when placed in Fe-deficient conditions, disparities in growth status between the overexpressed and regular apple calli were observed, with the genetically modified calli showing superior growth performance compared to the natural variant. Furthermore, lines OE-1, OE-6, and OE-8 showed elevated activities of POD, SOD, CAT, and FCR, as well as increased Fe^2+^ levels compared to the standard apple calli (Supplementary Figure S5). The levels of endogenous hormones, such as ABA and IAA, also increase in lines OE-1, OE-6, and OE-8. Moreover, bromocresol violet staining showed that *MhPYL4*-A transgenic calli released more H^+^ into the nearby medium when exposed to Fe-deficient conditions comparison to the typical control counterpart (Supplementary Figure S5). Consequently, it can be inferred that the implementation of *MhPYL4*-A contributed to enhancing the resilience of the apple callus to Fe scarcity.

## 4. Discussion

Plants experience various abiotic stressors, such as water scarcity, soil salinity, and nutrient imbalances, during their growth and development [23]. Fe deficiency is a widespread problem that can cause substantial leaf yellowing and decreased vigor in fruit trees, ultimately affecting the quality and quantity of apples produced [24]. When facing Fe deficiency, plants modulate the expression of stress response genes to alleviate its effects [25].

Transcription factors play crucial roles in regulating various biological processes, including stress responses, by binding to cis-acting elements in gene promoters. Positioned at the apex of the inhibitory pathway, PYR/PYLs control ABA signaling by inhibiting PP2Cs. During abiotic stress, such as Fe deficiency, plants rapidly produce abscisic acid (ABA), which then binds to PYR/PYL receptors, thereby facilitating their interaction with PP2Cs and relieving the activating SnRK2. This increase in SnRK2 activity leads to the phosphorylation of bZIP transcription factor phosphorylation, enabling interaction with ABA-related components and the subsequent activation of ABA-responsive genes.

The gene family PYL has been studied in various organisms, including *Arabidopsis* [26], grape [27], strawberry [28], and rice [29], among others. ABA is associated with sugar metabolism, and AS6 increases internal ABA levels in fruits, leading to enhanced sugar metabolism rates [30]. Studies have shown that *Arabidopsis* has 13 members of this gene family, named PYL1~13, that encode soluble proteins with the characteristic START (STAR-related lipid transfer) structural domain. These genes are mainly located in the cytoplasm and nucleus [31]. Overexpression of *PYL3* in rice improved cold and drought tolerance in *Arabidopsis thaliana* [32]. *Arabidopsis* and rice plants with the pRD29A/*PYL9* gene exhibited increased survival rates and delayed leaf senescence under drought conditions, indicating that *PYL9* genes enhance drought tolerance and delay leaf senescence [33]. This demonstrates the diverse functions of PYL family members. By analyzing previous transcriptome data and using real-time quantitative PCR, *PYL4*, which is induced by Fe deficiency, was identified and cloned to investigate its role in Fe deficiency.

Fe deficiency impacts chlorophyll synthesis and chloroplast development, leading to interveinal chlorosis and decreased photosynthesis in developing leaves. Studies have shown that transgenic plants, like *Arabidopsis thaliana* and tobacco, demonstrate higher rates of leaf chlorosis compared to non-transgenic plants, indicating potential adaptations to moderate Fe levels [34,35]. Our research compared the levels of chlorophyll a, chlorophyll b, total chlorophyll, and carotenoids in transgenic and non-transgenic *Arabidopsis*. The findings revealed significantly elevated pigment contents in transgenic plants under Fe-deficiency conditions, highlighting the susceptibility of the photosynthetic system in non-transgenic plants to Fe deficiency. Moreover, Fe deficiency induces the production of reactive oxygen species in chloroplasts, specifically singlet oxygen species that oxidize carotenoids and modify gene expression for stress adaptation [36]. Interestingly, our results show that the carotenoid content in transgenic *Arabidopsis* was notably lower than in non-transgenic *Arabidopsis*, indicating a lesser adaptability of transgenic plants to Fe-deficiency stress.

The H^+^-ATPase pumping system is capable of lowering soil pH by secreting H^+^ through the root epidermis, which facilitates the reduction of insoluble Fe^3+^ to soluble Fe^2+^ in the rhizosphere, characterizing the influence of species or genotype on Fe uptake effectiveness [37]. The functionality of FRO2 is dependent on the acidic pH and the flux of protons across the plasma membrane [38]. Simultaneously, under stress situations, significant quantities of ROS accumulate, which trigger the synthesis of a number of important antioxidant enzymes. For example, SOD, CAT, and POD remove reactive oxygen species (ROS) from the plant body and decrease the degree of damage to the membranes, thus enhancing the plant’s stress capacity [39]. In our study, under iron-deficiency stress, the enzyme activities (SOD, CAT, and POD) in transgenic Arabidopsis and overexpressed apple calli were significantly lower than in the corresponding wild-type plants, indicating that the wild-type plants had better antioxidant activity and reduced the accumulation of ROS radicals. Finally, Fe^3+^ can enter into various organelles and organs to be utilized only after it is reduced to Fe^2+^ by the enzyme ferric Fe reductase (FCR enzyme). Consequently, the FCR activity is closely correlated with the Fe content in the environment, and when the environment is Fe-scarce, the activity of Fe chelate reductase on the plasma membrane of the cell is significantly enhanced [40]. We assayed FCR activity in *Arabidopsis* and apple calli, showing that, under stress conditions, FCR activity in transgenic *Arabidopsis* and overexpressed apple calli was significantly lower than that in the wild-type control, which is inconsistent with the findings of Chen et al. [41]. The results indicate that transgenic plants can decrease Fe^3+^ reduction to reduce Fe utilization by reducing FCR activity.

The significant role of ABA in responding to abiotic or biotic stressors cannot be underestimated. ABA is recognized as a crucial hormonal cue triggered by various unfavorable environmental conditions, such as low temperatures and water scarcity [41]. Our findings suggest that there was minimal variation in ABA and IAA levels in apple calli overexpressing the *MhPYL4* gene under standard growth conditions. The IAA content in these calli was lower than that in the wild type when exposed to Fe-deficiency stress, while the ABA content was higher, showing a tendency to increase with prolonged Fe-deficiency stress exposure. These results indicate that overexpression of *MhPYL4* reduces the effectiveness of antioxidant enzymes and disrupts Fe reduction, worsening Fe deficiency.

## 5. Conclusions

In total, we demonstrated that *MhPYL4* plays a key role in Fe-deficiency stress in *Arabidopsis* and apple calli and can diminish the tolerance of Fe-deficiency stress by curtailing chlorophyll synthesis, decreasing antioxidant enzyme activity, and attenuating Fe-reducing properties.

## Data Availability

Data will be made available on request.

## Supplementary Materials

The following supporting information can be downloaded at: https://www.mdpi.com/article/10.3390/plants13162317/s1, Figure S1: Information on the *MhPYL4* protein sequence. (A) Electrophoresis of PCR products for cloning of *MhPYL4*. (B) Electrophoresis images of pRI101-*MhPPYL4* PCR amplification. (C) Electrophoresis of PCR products for cloning of *MhPYL4-A*. (D) Electrophoresis images of pRI101-*MhPPYL4-A* PCR amplification. (E) Identification of transgenic MhPYL4 *Arabidopsis* plants. (F) Identification of transgenic *MhPYL4* apple calli. (G) Identification of transgenic *MhPYL4-A* apple calli. Figure S2: Protein sequence analysis of *MhPYL4* gene in *Malus halliana* and the protein of other species. Figure S3: Phylogenetic analysis of this protein of *MhPYL4* from *Malus halliana* and other species. The number at the branch of the evolutionary tree indicates the confidence of the branch; the larger the value, the higher the reliability. Figure S4: Some important *cis*-acting regulatory elements in the upstream regulatory sequences of *MhPYL4*. Figure S5: Overexpression of *MhPYL4 Arabidopsis thaliana* for resistance to Fe-deficiency stress. (A) Chlorophyll a. (B) Chlorophyll b. (C) Carotenoids. (D) Chlorophyll a+b. (E) O_2_^−^ content. (F) H_2_O_2_ content. Table S1. List of primers for real-time quantitative PCR analysis. Table S2. Primers used in this study. Table S3. Physical and chemical properties of *MhPYL4* gene.
