# Supplementary material for: Functional Identification of MhPYL4 Involved in Iron-Deficiency Stress in Malus Halliana Koehne
Source: Plants (Basel). 2024 Aug 20;13(16):2317. doi: 10.3390/plants13162317 (PMC11360065; doi:10.3390/plants13162317)
Supplement: Supplementary file 1 [file plants-13-02317-s001.zip › plants-3111875-supplementary.pdf]

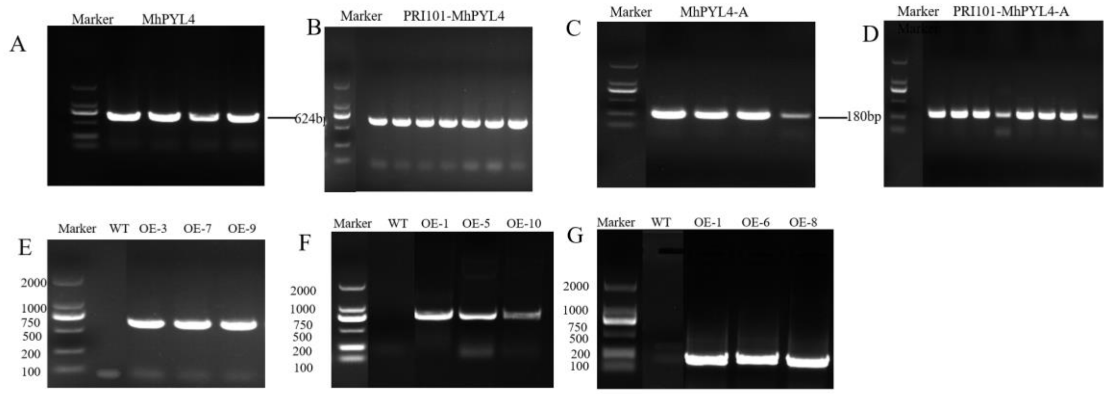

Supplementary Figure S1 Information on the *MhPYL4* protein sequence. (A) Electrophoresis of PCR products for cloning of *MhPYL4*. (B) Electrophoresis images of pRI101-*MhPPYL4* PCR amplifcation. (C) Electrophoresis of PCR products for cloning of *MhPYL4-A*. (D) Electrophoresis images of pRI101-*MhPPYL4-A* PCR amplifcation. (E) Identification of transgenic MhPYL4 *Arabidopsis* plants. (F) Identification of transgenic *MhPYL4* apple calli. (G) Identification of transgenic *MhPYL4-A* apple calli.

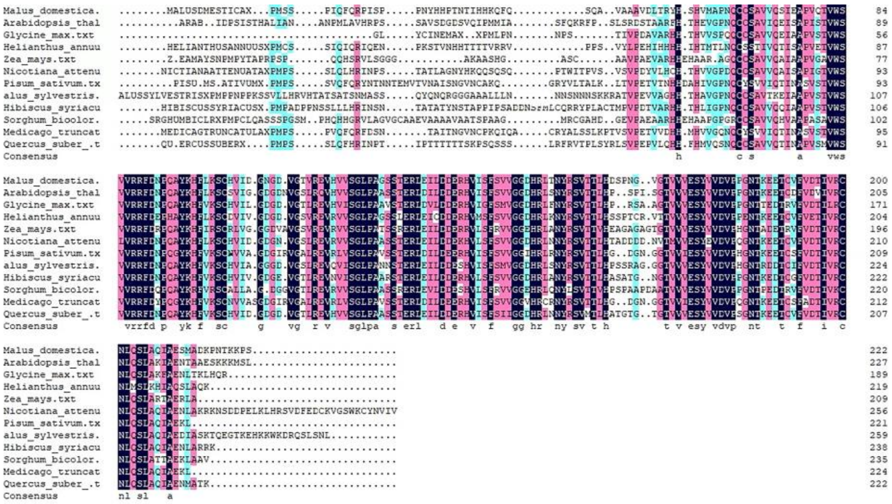

Supplementary Figure S2 Protein sequence analysis of *MhPYL4* gene in *Malus halliana* and the protein of other species

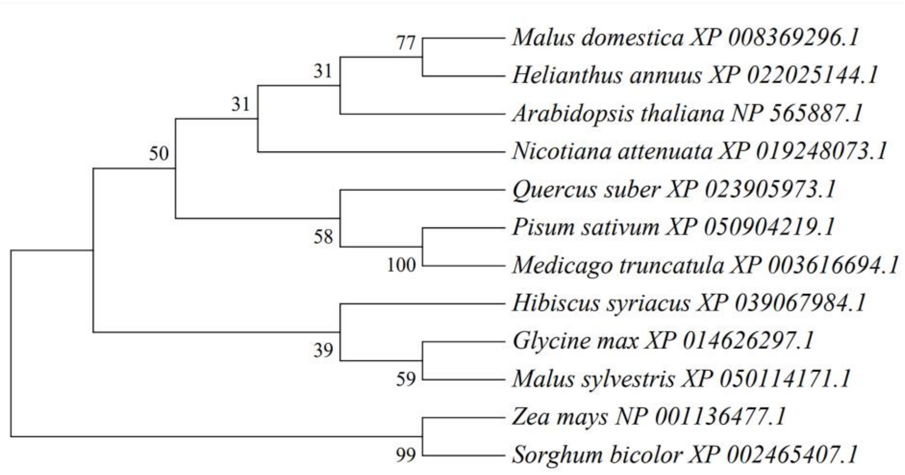

Supplementary Figure S3 Phylogenetic analysis of this protein of *MhPYL4* from *Malus halliana* and other species The number at the branch of the evolutionary tree indicates the confidence of the branch,the larger the value, and the higher the reliability.

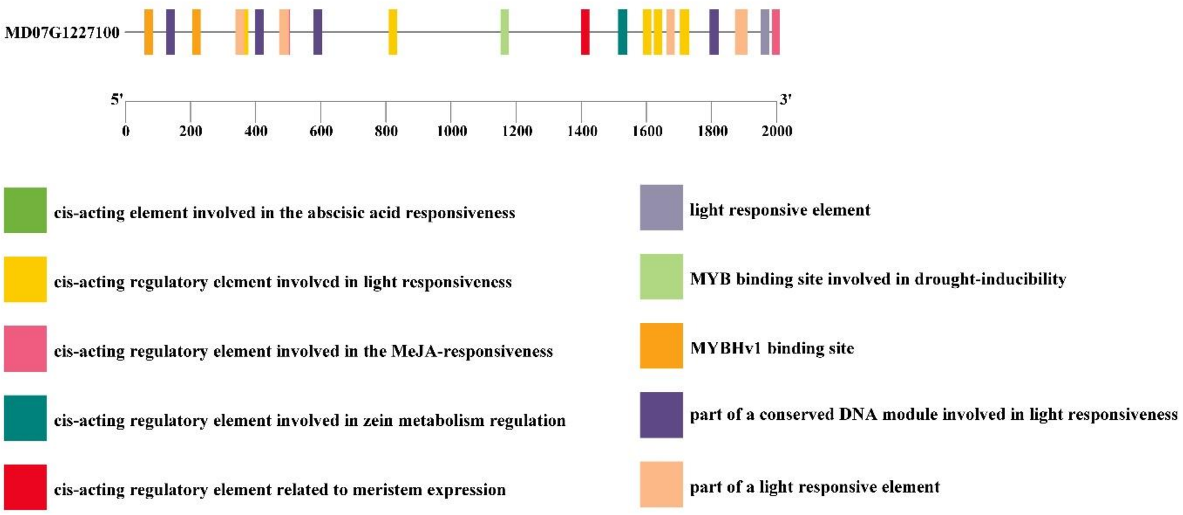

Supplementary Figure S4. Some important *cis*-acting regulatory elements in the upstream regulatory sequences of *MhPYL4*

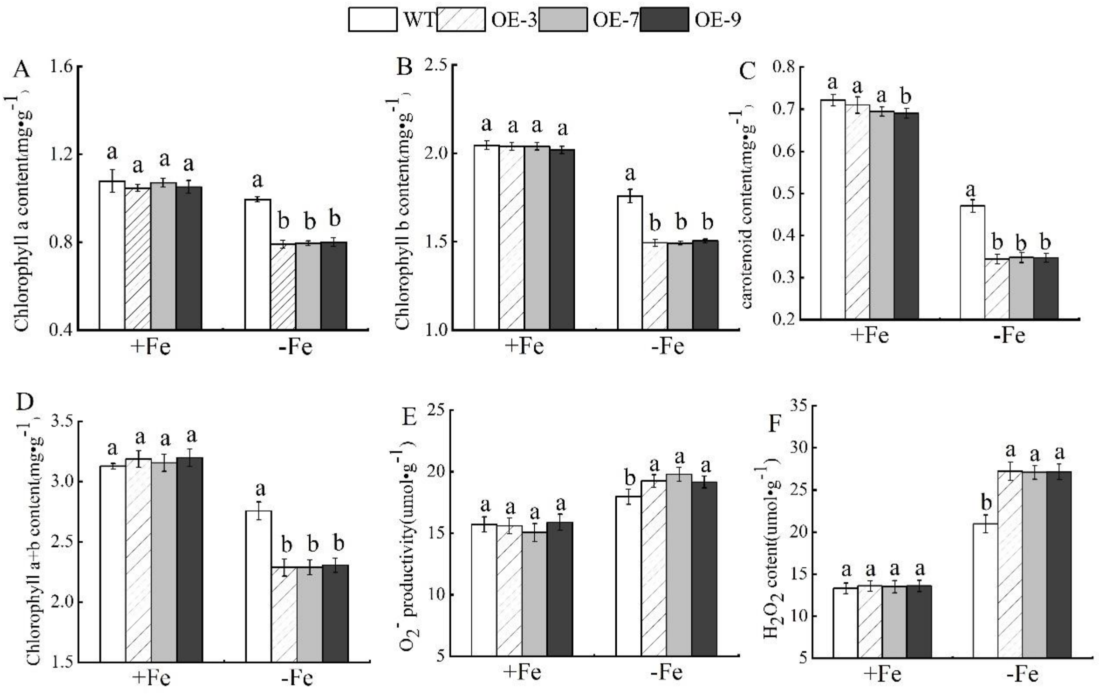

Supplementary Figure S5. Overexpression of *MhPYL4 Arabidopsis thaliana* for resistance to Fe deficiency stress. (A) Chlorophyll a. (B) Chlorophyll b. (C)carotenoids. (D) Chlorophyll a +b.(E) O_2^- content. (F) H_2O_2 content.

Supplementary Table S1. List of primers for real time quantitative PCR analysis

Gene name	Primer sequence (5'-3')	
	Forward primer	Reverse primer
MdPYL1	AACGAGTTCGACGAGTTGAAGAGC	CGCACCACCGACCACACG
MdPYL2	ATACCAGAAGGGAACACCGAGGAG	CAGATTCGCCGCCATAGCCATC
MdPYL4	GCAGTCGCAGCCGCAGTC	GACAGGTGCCTCGATGGATTGC
MdPYL6	TCCGTCCTCCGCCAGTTCG	TGCTCCCTATGCCTCCGTTCC
MdPYL8	GGAATAGGAATGGAGGCGGAGGAG	ATGGTCGTTAAGGTCGTGCTTGTG
MdPYL9	AGTACATACGGAGGCACCACAGG	CTCACCAACGACCACACGAGATG

Supplementary Table S2. Primers used in this study.

Gene name	Primer sequence (5'-3')	
	Forward primer	Reverse primer
MhPYL4	ATGTCTTCACCAATCCAGTTTC	TCATGAGGGTTTCTTGGTGTT
MhPYL4-PRI101	CATATGCCCGTCGACCCCGGGATGTC	TCAGAATTCGGATCCGGTACCTCATGA
	TTCACCAATCCAGTTTC	GGGTTTCTTGGTGTT
MhPYL4-A	ATGTCTTCACCAATCCAGTTTC	TCATGAGGGTTTCTTGGTGTT
MhPYL4-A-PRI101	TCAGAATTCGGATCCGGTACCTCAT	CATATGCCCGTCGACCCCGGGTCATG
	GAGGGTTTCTTGGTGTT	AGGGTTTCTTGGTGTT
MhPYL4-35seGFP	GGACAGGGTACCCGGGGATCCAT	CACCATGGTACTAGTGTCGACTG

	GTCTTCACCAATCCAGTTTCAA	AGGGTTTCTTGGTGTTTGGC

Supplementary Table S3. Physical and chemical properties of *MhPYL4* gene

Gene	Amino acids	Molecular weight (KD)	Theoretical pI	Positive residues	Negative residues	Aliphatic index	Instability index	Grand average of hydropathicity
MhPYL4	207	22.74	6.44	17	20	80.34	47.65	-0.293
